# Activation of the Keap1-Nrf2 pathway by specioside and the *n*-butanol extract from the inner bark of *Tabebuia rosea* (Bertol) DC

**DOI:** 10.12688/f1000research.26901.3

**Published:** 2020-12-02

**Authors:** Sandra Catalina Garzón-Castaño, Francisco Javier Jiménez-González, Luz Angela Veloza, Juan Carlos Sepúlveda-Arias

**Affiliations:** 1 Grupo Infección e Inmunidad, Facultad de Ciencias de la Salud, Universidad Tecnológica de Pereira, Pereira, Colombia; 2 Grupo de Biomedicina, Facultad de Medicina, Fundación Universitaria Autónoma de las Américas, Pereira, Colombia; 3 Grupo Polifenoles, Facultad de Tecnologías, Universidad Tecnológica de Pereira, Pereira, Colombia

**Keywords:** Tabebuia rosea, specioside, Bignoniaceae, extracts, Nrf2, antioxidant agents

## Abstract

**Background:** A large number of chemical compounds exert their antioxidant effects by activation of key transcriptional regulatory mechanisms, such as the transcription factor Nrf2. The aim of this study was to evaluate the activation of the Keap1-Nrf2 pathway by both the *n*-butanol extract obtained from the inner bark of *Tabebuia rosea* (Bertol) DC and specioside isolated from this extract.

**Methods:** The antioxidant activity of the extract and specioside isolated from the inner bark of *T. rosea* were evaluated using the oxygen radical absorbance capacity (ORAC) and the 2,2-diphenyl-1-picrylhydrazyl radical scavenging activity (DPPH) techniques, whereas their effects on the viability of HepG2 cells was determined using the 3-(4,5-dimethylthiazol-2-yl)-2,5-diphenyltetrazolium bromide (MTT) method. The effects of the compound and the extract on activating the Keap1-Nrf2 pathway were evaluated using a Nrf2 Transcription Factor Assay kit. Induction of the Nrf2-mediated antioxidant response genes *HMOX-1* and *NQO1* was evaluated by real-time PCR. The protective effects against H _2_O _2_-induced oxidative stress in HepG2 cells was determined as the percent protection using the MTT method.

**Results: **Both the *n*-butanol extract and specioside exhibited activity at low concentrations without affecting cellular viability, since the cell viability was greater than 80% after 24 hours of exposure at each tested concentration. In addition, Nrf2 dissociated from Keap1 after treatment with the *n*-butanol extract at a concentration of 0.25 µg/mL after 4 hours of exposure. An increase in the Nrf2 level in the cytoplasm after 4 hours of exposure to 2 μM specioside was observed. Nrf2 levels stabilized in the nucleus 12 hours after stimulation with both specioside and the extract. After 6 hours of stimulation, both the extract and specioside induced the expression of *HMOX-1 *and *NQO1*.

**Conclusion:** The *n*-butanol extract from the inner bark of *T. rosea* and specioside produced protective effects against H _2_O _2_-induced oxidative stress in HepG2 cells.

## Abbreviations

AAPH, 2,2’-Azobis(2-amidinopropane)dihydrochloride; ALA, alpha-lipoic acid; CUR, curcumin; ORAC, oxygen radical absorbance capacity; DPPH, 2,2-diphenyl-1-picrylhydrazyl radical scavenging activity; MTT, 3-(4,5-dimethylthiazol-2-yl)-2,5-diphenyl tetrazolium bromide; CHAL, 2-trifluoromethyl-2ʹ-methoxychalcone

## Introduction

Over the years, plants have been used to treat several diseases, are considered an important source of biologically active natural products, and many have been used for the synthesis of various drugs. The natural products present in nature have great diversity in terms of their chemical structures and physicochemical properties, in addition to their biological activities. The *Tabebuia* genus belongs to the Bignoniaceae family, which includes more than 800 species and is considered as one of the most abundant family of plants in the neotropics ^1^. The genus *Tabebuia* is distributed from Mexico to South America and has been used in traditional medicine. Several bioactive molecules such as saponins, quinones, tannins and alkaloids ^2, 3^ found in *Tabebuia* species have anti-inflammatory and antioxidant activities ^4– 6^. There is a wide distribution of the species *Tabebuia rosea* throughout Colombia, which has motivated the study of its therapeutic properties. The antioxidant capacity of extracts obtained from *T. rosea* has not been widely studied, although this activity has been demonstrated in other species of the *Tabebuia* genus.

Oxidative stress is the result of an imbalance between the production of reactive oxygen species (ROS) and the cellular antioxidant capacity, inducing oxidative damage, which plays a role in the development of premature aging, chronic diseases and cancer ^7– 11^. In addition, oxidative stress contributes to pathogenesis in many neurodegenerative diseases, such as Parkinson's, Alzheimer's, Huntington's and amyotrophic lateral sclerosis, where increases in oxidative markers have been found ^12, 13^. Cells respond to oxidative stress through various defense mechanisms, such as the elimination of free radicals and the generation of antioxidant molecules mediated by the transcription factor Nrf2 (nuclear factor erythroid 2-related factor 2). Nrf2 is a redox-sensitive transcription factor that plays a major role in cell defense against oxidative stress. Nrf2 belongs to a family of basic proteins with a leucine zipper domain (bZip) ^14^. Under normal conditions, Nrf2 is localized in the cytoplasm and is inhibited by the Keap1 protein (Kelch ECH associating protein 1) and therefore degraded in the proteasome. In the presence of oxidative stress, Nrf2 translocates to the nucleus. Once there, it binds to the ARE site and induces the expression of antioxidant enzymes such as NAD(P)H quinone oxidoreductase (NQO1) and heme oxygenase 1 (HMOX-1). This response is important to modulate the homeostatic balance of the cells ^15– 20^. A large number of molecules activate transcriptional regulatory mechanisms to induce their antioxidant activity.

Natural product research, guided by ethnopharmacological knowledge, has made significant contributions to drug innovation by providing new chemical structures and understanding of their mechanisms of action. Considering the potential health benefits and the possible pharmacological effects of extracts obtained from *T. rosea*, its abundance in Colombia and the few investigations regarding its antioxidant properties and the molecular mechanisms involved in its activity, the aim of this study was to evaluate the mechanism responsible for the *in vitro* antioxidant activity of the *n*-butanol extract obtained from the inner bark of *T. rosea* (Bertol) DC.

## Methods

### Plant material, extract preparation and specioside isolation

The inner bark of *T. rosea* (Bertol) DC was collected at the Universidad Tecnológica de Pereira campus in April 2011. The plant was identified at the Colombian National Herbarium (voucher no. COL 582577). The collection and processing of the material were covered by collection permission number 1133/2014 issued by the National Environmental Licensing Authority (ANLA) of Colombia.

Extracts were obtained as previously described ^21, 22^. Plant material (2 Kg) was dried and macerated in analytical grade methanol for 48 hours. This was followed by homogenization, filtration, and concentration under vacuum using a vacuum rotary evaporator (Heidolph, Laborota model) at 40°C to obtain the crude extract (294.3 g, yield 14.7%). The crude extract was dissolved in 400 mL of distilled water and underwent liquid–liquid extraction with increasing polarity solvents: *n*-hexane, chloroform (CHCl _3_), ethyl acetate (EtOAc), and *n*-butanol (all solvents were of analytical grade). Each extract was vacuum dried by a vacuum rotary evaporator. Endotoxin levels in the extracts were assayed using the Limulus Amebocyte Lysate Test (E-Toxate kit, Sigma Chemical Co., Saint Louis, MO, USA, Cat No. ET0200-1KT). The *n*-butanol extract was kept refrigerated at 4°C in an amber tube protected from light, heat, air and moisture. For each of the biological assays, the extract was dissolved in 0.1% DMSO (dimethyl sulfoxide, Merck, Darmstadt, Germany, Cat No. 1029521000).

**Figure 1.**
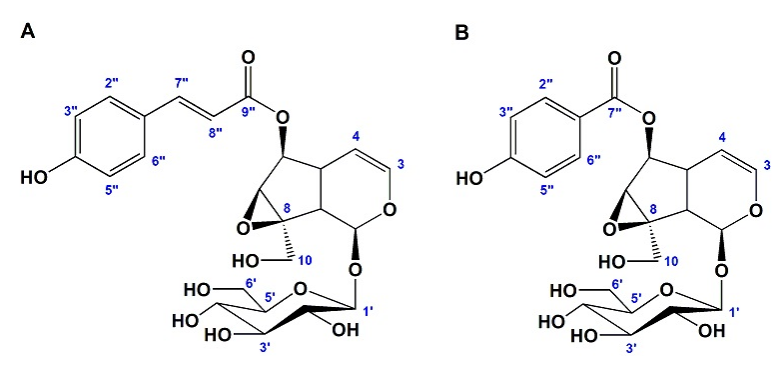
Chemical structure of specioside ( **A**) and catalposide ( **B**).

The butanolic extract was concentrated with rotary evaporation under reduced pressure, obtaining a dark brown extract (12.5 g, yield 4.25%). The butanolic extract (8.0 g) was subjected to separation by column chromatography (CC) on Diaion® HP-20 (Mitsubishi Chemical Corp.), with a water-isopropanol elution gradient (90:10 to 10:90), obtaining subfractions A-J. Tr-1 (25.9 mg) was isolated from subfraction D with a semipreparative HPLC-DAD system (Hitachi-Merck) in reversed-phase (LiChrocart 250-10, LiChrospher 100; 10 µm, Merck) by isocratic elution with H _2_O-ACN (70:30% v/v) containing 1% v/v CH _3_COOH. Specioside (Tr-1, Figure 1) was obtained as a dark brown amorphous solid (m.p. 142-162°C). The FTIR spectrum displayed absorption bands attributable to carbonyl groups (ν _C=O_ 1698 cm ^-1^), hydroxyl groups (ν _O-H_ 3383 cm ^-1^), and aromatic rings (ν _C=C_ 1605 cm ^-1^).

Full assignments from the ^1^H and ^13^C NMR spectra were made through the use of ^1^H- ^1^H COSY, HSQC and HMBC experiments. All the experiments were performed on a 400 MHz Agilent spectrometer (125.6 MHz for ^13^C); using deuterated methanol as solvent. The ^1^H NMR spectrum showed two olefinic protons at δ _H_ 6.37 (H-3, dd) and δ _H_ 4.98 (H-4, dd), characteristic of the iridoid nucleus. This structure was confirmed by correlations shown in the HMBC spectrum with carbons at δ _C _140.95 (C-3) and δ _C_ 101.50 (C-4). In addition, two olefinic protons at δ _H_ 7.67 (1H, d, *J* = 16.0 Hz, H-7”) and δ _H_ 6.38 (1H, d, *J* = 15.9 Hz, H-8”) suggested the presence of an *E* configuration, which is characteristic of a *p*-coumaroyl skeleton. The *p*-coumaroyl structure was confirmed by the observation of two signals at δ _H_ 7.48 (2H, d, *J* = 8.7 Hz, H-2”, H-6”) and δ _H_ 6.81 (2H, d, *J* = 8.7 Hz, H-3”, H-5”), characteristic of an AA’XX’ system; these data were confirmed by the ^13^C NMR spectrum, which exhibited eight carbon signals, including carbonyl carbon δ _C_ 164.49 (C-9’’), which was attributed to the *p*-coumaroyl ester. The presence of anomeric protons at δ _H_ 4.79 (1H, d, *J* = 7.9 Hz, H-1’), and methine signals at δ _H_ 3.42–3.23 (4H, m) are characteristic of a sugar moiety. Analysis of the 1D and 2D NMR spectra in addition to comparisons with literature data for glucoside analogs suggested that the saccharide portion was a glucose moiety. Characteristic ^1^H NMR, ^13^C NMR, COSY, HSQC and HMBC spectra are supplied as *Extended data* ^23^.

### Oxygen radical absorbance capacity (ORAC)

The oxygen radical absorbance capacity was determined using the method described by Ou *et al.* with some modifications ^24^. 2,2’-Azobis(2-amidinopropane)dihydrochloride (AAPH) and sodium fluorescein stock solutions were prepared in a 75 mM, pH 7.0 phosphate buffer solution. Thirty-one microliters of each sample was diluted in 187 µL of fluorescein (120 nM) and incubated at 37°C for 10 min. The reaction was started by the addition of 31 µL of AAPH (143 mM) to reach a final volume of 249 µL per well. The extract was evaluated at concentrations of 0.25, 0.5, 1 and 2 µg/mL. Specioside and catalposide ^25^ (Sigma Chemical Co., Saint Louis, MO, USA, Cat No. SMB00094-1MG) as well as the controls (α-lipoic acid ^26, 27^, curcumin ^28, 29^ and quercetin ^30, 31^) were evaluated at concentrations of 0.5, 1, 2 and 4 µM. A Trolox® standard curve was prepared. Changes in fluorescence were measured with a Varian Cary Eclipse 1.1 fluorescence spectrophotometer at 2 min intervals for 120 min with emission and excitation wavelengths of 515 and 493 nm, respectively. The excitation slit width was 5 nm, as was the emission slit width ^32, 33^. The antioxidant capacity was calculated as the area under the curve (AUC) ^34^ and is expressed as µmol Trolox® equivalents per liter (µmol TE/L).

### 2,2-Diphenyl-1-picrylhydrazyl radical scavenging activity (DPPH)

The antioxidant activity of the extract and compounds was also evaluated by the DPPH method using the methodology described by Brand-Williams *et al.* with some modifications ^35^. Thirty microliters of samples and controls were prepared at concentrations ranging from 0.25 to 2 µg/mL and 0.5 – 4 µM, respectively, and mixed with 2 mL of a methanol solution of DPPH (20 μg/mL DPPH, 5 × 10 ^−5^ mol/L); each mixture was agitated and kept in the dark for 30 min at RT. The absorbance was measured at 517 nm in a Shimadzu UV–1700 spectrophotometer. Ascorbic acid (5 – 200 μg/mL) was used for the standard curve. Each experiment was repeated three times, and the antioxidant capacity was calculated as the percent inhibition ^36^.

### Cell culture

The human hepatocarcinoma cell line (HepG2; ATCC; CRL-11997) was purchased from the American Type Culture Collection (ATCC, Rockville, MD, USA) and cultured with Dulbecco’s modified Eagle’s medium (DMEM) supplemented with Glutamax (GIBCO/BRL, USA, Cat No. 10564-011) and 10% heat-inactivated FBS (GIBCO, Cat No. 16140071), 200 µg/mL penicillin, 200 µg/mL streptomycin, 400 µg/mL neomycin (GIBCO, Cat No. 15640-055), 5 µg/mL amphotericin and 1 mM sodium pyruvate (all from Sigma Chemical Co., Saint Louis, MO, USA, Cat Nos. A9528-50MG and S8636-100ML, respectively). Cells were maintained at 37°C in a 5% CO _2_ atmosphere.

### Cell viability test

The viability of HepG2 cells in the presence of the extracts and compounds was tested using the MTT (3-(4,5-dimethylthiazol-2-yl)-2,5-diphenyl tetrazolium bromide) method ^37^. Cells (5×10 ^4^ cell/well) were treated with the extracts (0.25, 0.5 and 1 µg/mL) and compounds - 0.5, 1 and 2 µM specioside, catalposide and controls: α-lipoic acid (ALA), curcumin (CUR) and 2-trifluoromethyl-2ʹ-methoxychalcone ^38^ (CHAL), diluted in DMSO and incubated for 24 hours. After treatment, the medium was discarded, and 200 μL of DMEM containing 0.5 mg/mL MTT (Sigma Chemical Co., Saint Louis, MO, USA, Cat No. M2128-500MG) was then added to each well. The plates were incubated for 4 hours at 37°C in a 5% CO _2 _atmosphere. The medium was discarded, and 100 μL of DMSO was then added to solubilize the formazan crystals. The absorbance was measured with an ELISA microplate reader at 492 nm (ELx800; BioTek Instruments Inc., USA). The percent viability was calculated based on the nontreated control. Three independent assays were performed, each in triplicate.

### Nrf2 nuclear activation

A total of 1×10 ^6^ HepG2 cells/well were cultured in DMEM. The medium was discarded, and the cells were exposed to the extract (0.25 or 1 µg/mL), compounds or controls (0.5 or 2 µM) for 0, 4, 12 or 24 hours. After exposure, cells were harvested and used for the simultaneous extraction of nuclear and cytosolic proteins following the specifications included in the Nuclear Extraction Kit (Abcam, Cambridge, UK, ab113474). Total protein was quantified using the BCA Protein Quantification Kit (Abcam, Cambridge, UK, ab102536). Nrf2 was detected by using the Nrf2 Transcription Factor Assay Kit (Colorimetric, Abcam, Cambridge, UK, ab207223) following the manufacturer’s instructions. The absorbance of each well was measured at 450 nm in an ELx800 BioTek microplate reader.

### qRT-PCR assays

HepG2 cells (3×10 ^5^ cells/well) were treated with extract (0.25 or 1 µg/mL), compounds or controls (0.5 or 2 µM) for 0, 4, 6 or 8 hours. After treatment, mRNA extraction was performed using the RNeasy Plus Mini Kit (Qiagen, Maryland, USA, Cat No. 74134). mRNA was quantified with a NanoDrop 2000c (Thermo Fisher Scientific, Waltham, MA). The expression of the *HMOX-1* and *NQO1* genes ^21, 39– 41^ was evaluated by RT-qPCR using predesigned TaqMan Gene Expression Assays (Hs01110250_m1 and Hs01045993_g1) and the TaqMan® RNA-to-CT ^TM^ 1-Step Kit (Applied Biosystems, Foster City, CA, Cat No. 4331182). The run method was holding at 48°C for 15 min, 95°C for 10 min and cycling (40 cycles) at 95°C for 15 sec and 60°C for 1 min. β-actin (Applied Biosystems, Foster City, CA, ref. 4325788) was used as an endogenous control. ****

### Protective effects of the extract and compounds

Hydrogen peroxide (H _2_O _2_) was employed as a stressor agent in order to evaluate the protective capacity of the extract (0.25 and 1 µg/mL) and compounds (0.5 and 2 µM). The controls used were ALA and CUR, since these compounds have been reported to have protective effect against oxidative stress mediated by Nrf2 ^28, 42^. Cells were grown to a density of 5×10 ^4^ cells/well in DMEM and incubated for 24 hours under a 5% CO _2_ atmosphere at 37°C. The medium was discarded, and the cells were exposed for 12 hours to different concentrations of the extract (0.25 or 1 µg/mL), compounds and controls (0.5 or 2 µM). Subsequently, the medium was discarded, and one of the plates was exposed to 0.98 mM H _2_O _2_ (previously determined concentration) and the second plate was used as a control. After 24 hours, 200 μL of MTT (0.5 mg/mL, Sigma) was added to both plates followed by incubation at 37°C for 4 hours. The medium was discarded, and 100 µL of 99.8% DMSO (Sigma) was added to solubilize the formazan crystals. The absorbance was measured in an ELISA microplate reader at 570 nm with correction at 630 nm (ELx800; BioTek Instruments Inc., USA). The percent inhibition was calculated.

### Statistical analysis

Each experiment was performed at least in duplicate. The results are expressed as the mean ± SD of at least three independent experiments. Statistical analysis was performed using the Kruskal-Wallis test, and a *p*-value less than 0.05 was considered statistically significant. The statistical tests were applied using GraphPad Prism, version 5.02 (GraphPad Software, San Diego, CA, USA).

## Results

### Antioxidant activity and cell viability after treatment with the *n*-butanol extract and pure compounds

The antioxidant activity of the *n*-butanol extract from the inner bark of *T. rosea*, the isolated compound specioside and the catalposide iridoid compound reported from the Bignoniaceae family were evaluated using the ORAC and DPPH techniques ^43^. The results showed that there was a tendency for the activity in the ORAC assay to be higher when the extract or compound concentration increased, displaying a concentration-dependent relationship, except for the control compound ALA. In addition, specioside, catalposide and the *n*-butanol extract displayed the best antioxidant activity, and this activity was significantly higher than that induced by ALA ( Table 1, *p* <0.05), whose percent DPPH inhibition was low (<25%). Specioside displayed the best antioxidant activity, followed by catalposide, quercetin, curcumin, α-lipoic acid and finally the *n*-butanol extract ( Table 1). The results from the MTT assay indicated that neither the *n*-butanol extract from the inner bark of *T. rosea* nor the pure compounds affected the viability of HepG2 cells, since the viabilities were all greater than 80% after 24 hours of exposure.

**Table 1.** Antioxidant activity of the *n*-butanol extract, specioside and catalposide using ORAC and DPPH techniques.

	ORAC (μmol TE/L)				DPPH (% Inhibition)			
Sample/Concentration (µg/mL)	0.25	0.5	1	2	0.25	0.5	1	2
**Specioside**	21.42 ± 1.62	20.95 ± 2.41	27.29 ± 3.49	**45.42 ± 6.93 ^b^**	15.13 ± 2.87	20.78 ± 0.53	23.04 ± 5.42	22.43 ± 6.38
**Catalposide**	18.08 ± 3.35	19.26 ± 3.19	16.99 ± 8.53	**35.09 ± 6.79 ^a^**	16.63 ± 2.75 ^c^	17.01 ± 1.04	15.08 ± 2.67	**16.94 ± 1.19 ^c^**
***n*-butanol extract**	13.13 ± 6.62	19.29 ± 3.75	24.92 ± 8.14	31.83 ± 4.92 ^a^	5.91 ± 4.17	4.64 ± 4.34	5.96 ± 3.88	5.53 ± 4.04
**α-Lipoic acid**	11.90 ± 0.97	6.97 ± 2.85	12.06 ± 6.11	12.60 ± 4.27	5.48 ± 2.38	6.19 ± 3.28	5.50 ± 4.00	4.97 ± 4.33
**Curcumin**	18.76 ± 3.12	27.85 ± 9.39	37.44 ± 7.12	66.90 ± 5.77	7.19 ± 6.52	8.42 ± 7.37	8.96 ± 8.65	12.19 ± 9.22
**Quercetin**	17.23 ± 3.32	19.69 ± 1.89	27.43 ± 5.79	52.53 ± 4.89	11.38 ± 5.88	10.51 ± 7.14	9.09 ± 8.20	11.76 ± 8.51

TE, Trolox equivalents.; ORAC, oxygen radical absorbance capacity; DPPH, 2,2-diphenyl-1-picrylhydrazyl radical scavenging activity. All experiments were carried out in triplicate. Data represent the mean ± SD. Kruskal Wallis, Dunn's multiple comparisons test. ^a^p<0.01, ^b^p<0.0001 compared with control (α-Lipoic acid). ^c^p<0.05 compared with extract and control (α-Lipoic acid).

### Effects of the *n*-butanol extract and pure compounds on the activation and nuclear translocation of Nrf2

**Figure 2.**
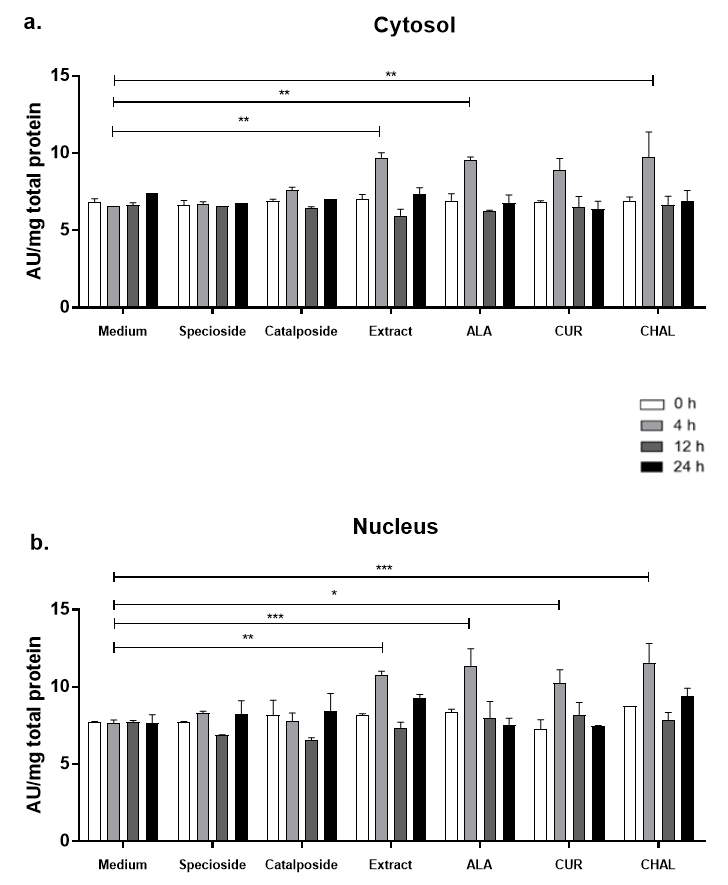
Nrf2 levels in cytosol ( **a**) and nucleus ( **b**) after 0, 4, 12 and 24 hours of exposure to 0.5 μM specioside, catalposide, controls and 0.25 µg/mL *n*-butanol extract. Kruskal-Wallis, Dunn's post hoc. * p<0.05, ** p<0.01, *** p<0.001. ALA, α-lipoic acid; CUR, curcumin; CHAL, 2-trifluoromethyl-2ʹ-methoxychalcone.

**Figure 3.**
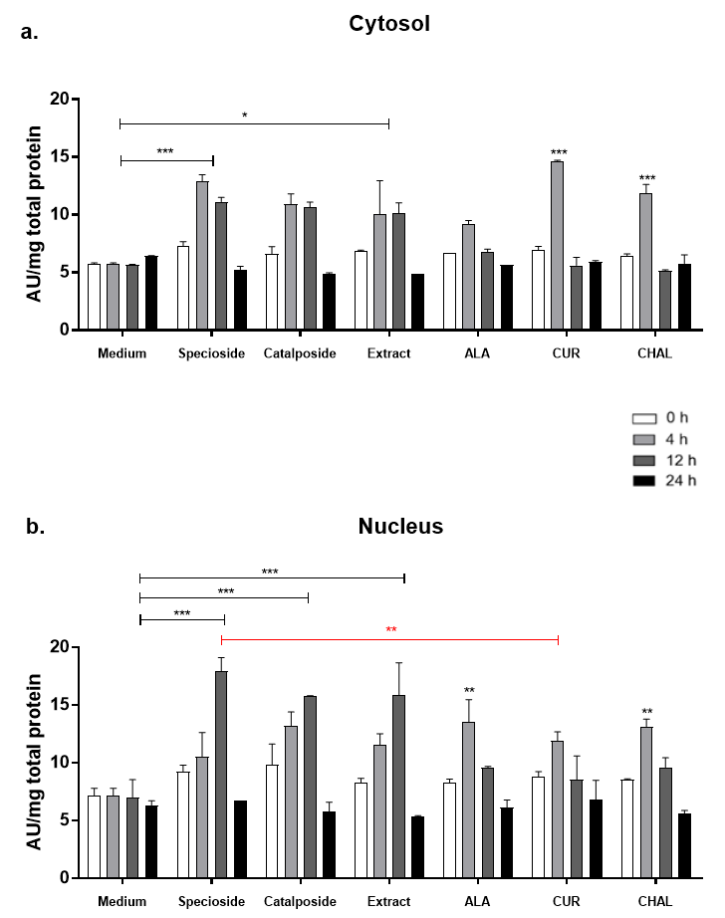
Nrf2 levels in cytosol ( **a**) and nucleus ( **b**) after 0, 4, 12 and 24 hours of exposure to 2 μM specioside, catalposide, controls and 1 µg/mL *n*-butanol extract. Kruskal Wallis, Dunn's post hoc. * p<0.05, ** p<0.01, *** p<0.001. ALA, α-lipoic acid; CUR, curcumin; CHAL, 2-trifluoromethyl-2ʹ-methoxychalcone.

Nrf2 detection enabled comparisons of the basal Nrf2 status in both the cytosol and the nucleus. It also allowed for comparison of their associated changes after exposure to the *n*-butanol extract (0.25 and 1 µg/mL) and the pure compounds specioside and catalposide (0.5 and 2 µM) in HepG2 cells. The results showed an increase in Nrf2 levels in the cytosol and nucleus after 4 hours of exposure ( Figure 2) to the extract (0.25 µg/mL) and the controls ALA, CUR and CHAL at the lowest concentration (0.5 µM), showing a significant difference (p <0.05). By increasing the concentration of the *n*-butanol extract to 1 µg/mL and the pure compounds and controls to 2 µM, increases in the Nrf2 levels were observed in all samples after 4 hours of exposure ( Figure 3), and this increase was maintained at 12 hours for specioside, catalposide and the *n*-butanol extract ( *p* <0.05). As shown in Figure 3b, the Nrf2 levels in the nucleus increased 4 and 12 hours after exposure to the pure compounds and the *n*-butanol extract. In addition, the level of the protein after 12 hours of exposure to specioside were significantly higher ( *p*<0.05) than that after exposure to the control CUR after 4 hours. This increase was measured in relation to the basal level (nonexposed cells).

### Effects of the *n*-butanol extract and pure compounds on the expression of HMOX-1 and NQO1

**Figure 4.**
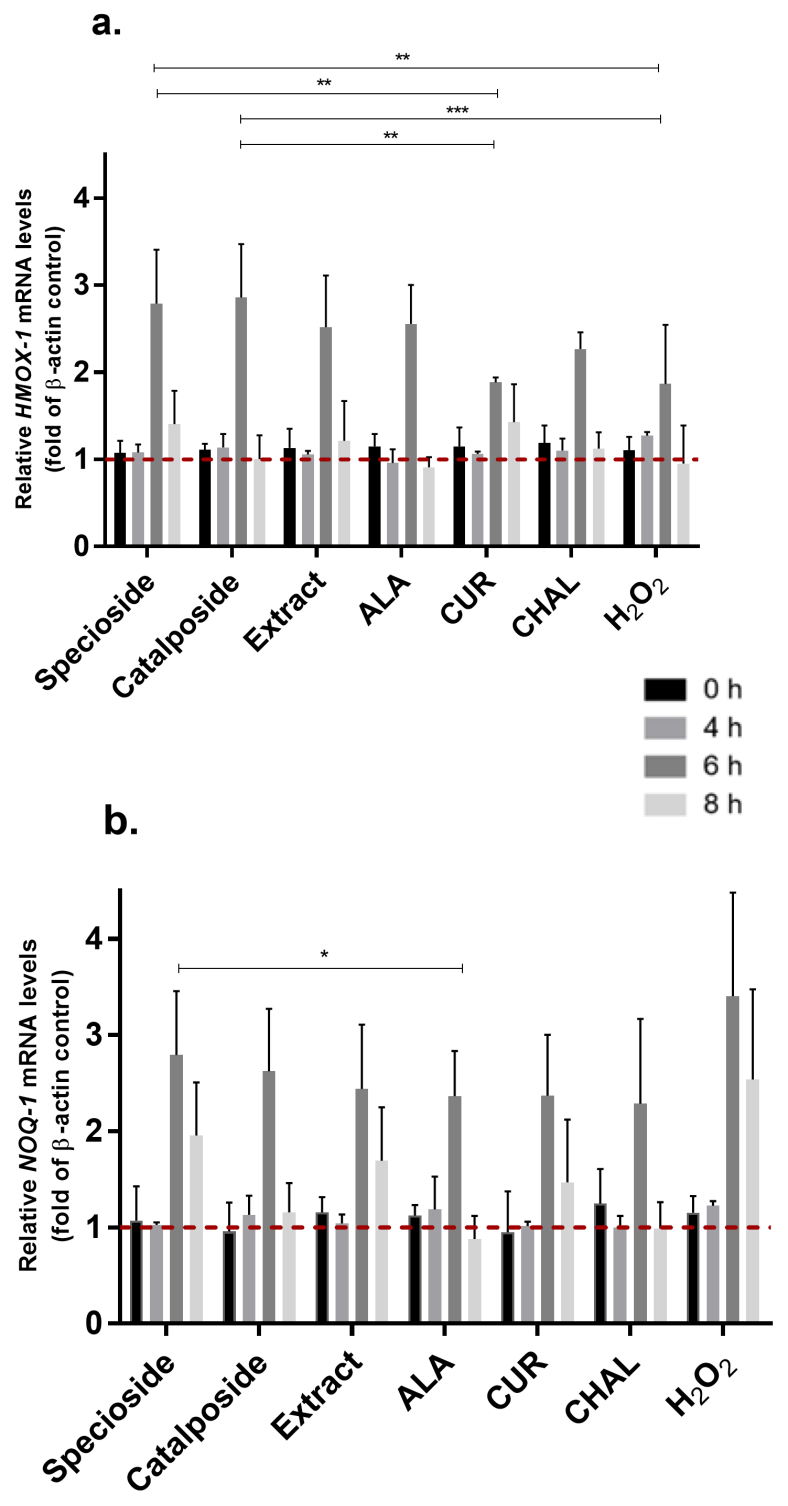
Relative *HMOX-1* ( **a**) and *NQO1* ( **b**) mRNA levels after 0, 4, 6 and 8 hours post-exposure to 0.5 µM (pure compounds), 0.25 µg/mL ( *n*-butanol extract) and induction of oxidative stress with 0.98 mM (H _2_O _2_). Kruskal Wallis Dunn's post hoc. * p<0.05, ** p<0.01, *** p<0.001. ALA, α-lipoic acid; CUR, curcumin; CHAL, 2-trifluoromethyl-2ʹ-methoxychalcone.

**Figure 5.**
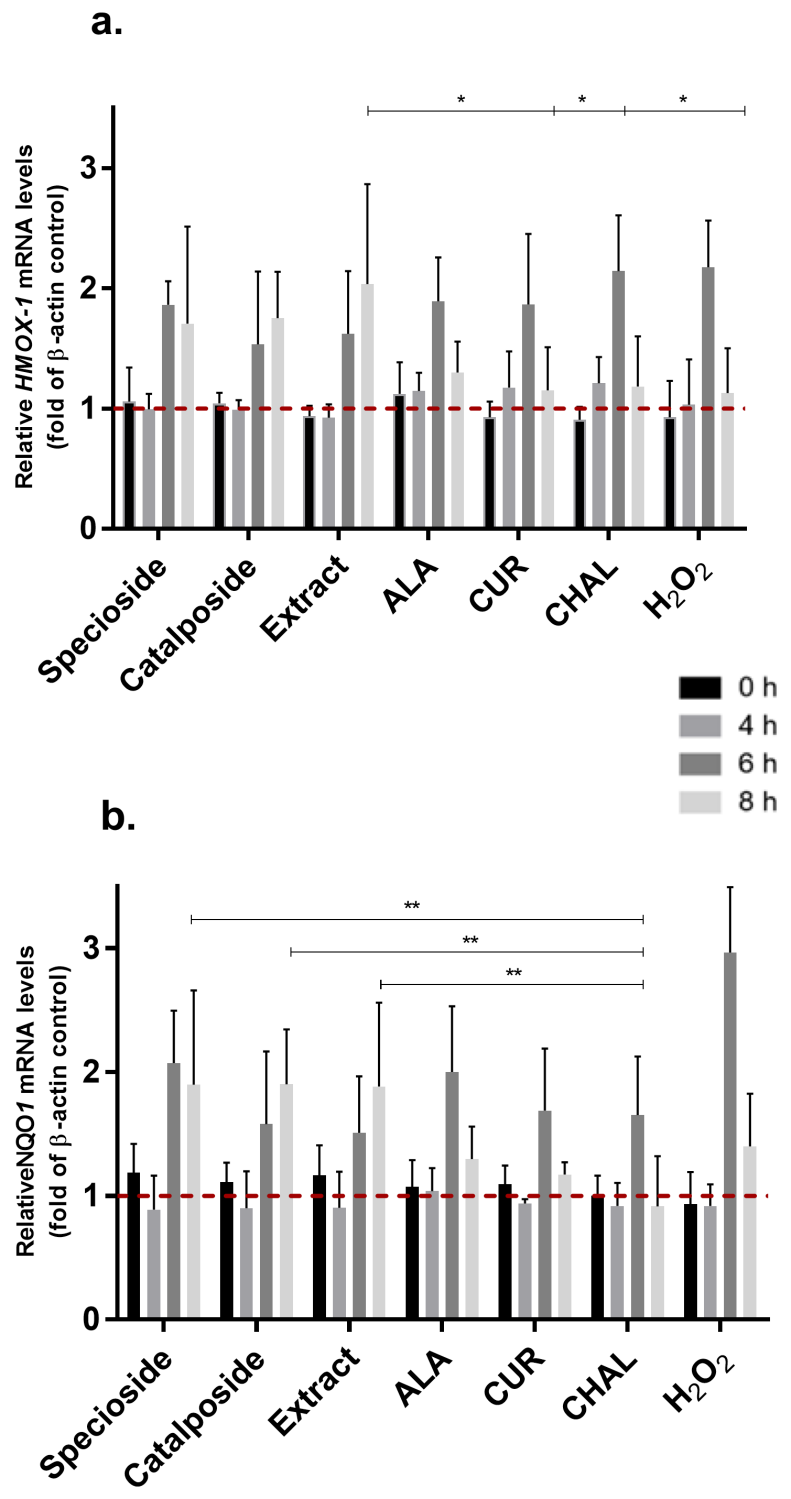
Relative *HMOX-1* ( **a**) and *NQO1* ( **b**) mRNA levels after 0, 4, 6 and 8 hours post-exposure to 2 µM (pure compounds), 1 µg/mL ( *n*-butanol extract) and 0.98 mM (H _2_O _2_). Kruskal Wallis, Dunn's post hoc * p<0.05, ** p<0.01, *** p<0.001. ALA, α-lipoic acid; CUR, curcumin; CHAL, 2-trifluoromethyl-2ʹ-methoxychalcone.

The levels of expression of the *HMOX-1* and *NQO1* genes were evaluated by qRT-PCR and quantified using the 2 ^-ΔΔCt^ method. The results indicate that the molecules specioside and catalposide (0.5 µM) and the *n*-butanol extract (0.25 µg/mL) increased the expression levels of *HMOX-1* (>1.5-fold change) and *NQO1* (>1.4-fold change) after 6 hours of exposure ( Figure 4). As shown in Figure 4a, the pure compounds significantly increased the expression levels of *HMOX-1* ( *p*<0.05) compared to the controls CUR and H _2_O _2_. The relative expression level of the *NQO1* gene increased significantly after treatment with specioside compared with the control ALA ( Figure 4b). At higher concentrations (1 µg/mL for the extract and 2 µM for the pure compounds and controls), the expression levels of *HMOX-1* and *NQO1* increased after 6 to 8 hours of exposure to specioside, catalposide and the *n*-butanol extract ( Figure 5). A significantly higher expression level of *HMOX-1* was observed after 8 hours of exposure to specioside, catalposide and the *n*-butanol extract compared with the controls (CUR, CHAL and H _2_O _2_, *p* <0.05, Figure 5a). The relative expression level of *NQO1* significantly increased after exposure to specioside, catalposide and the *n*-butanol extract compared to the control CHAL ( *p* <0.01, Figure 5b).

### Protective effects of the *n*-butanol extract and pure compounds against H _2_O _2_-induced oxidative stress

**Figure 6.**
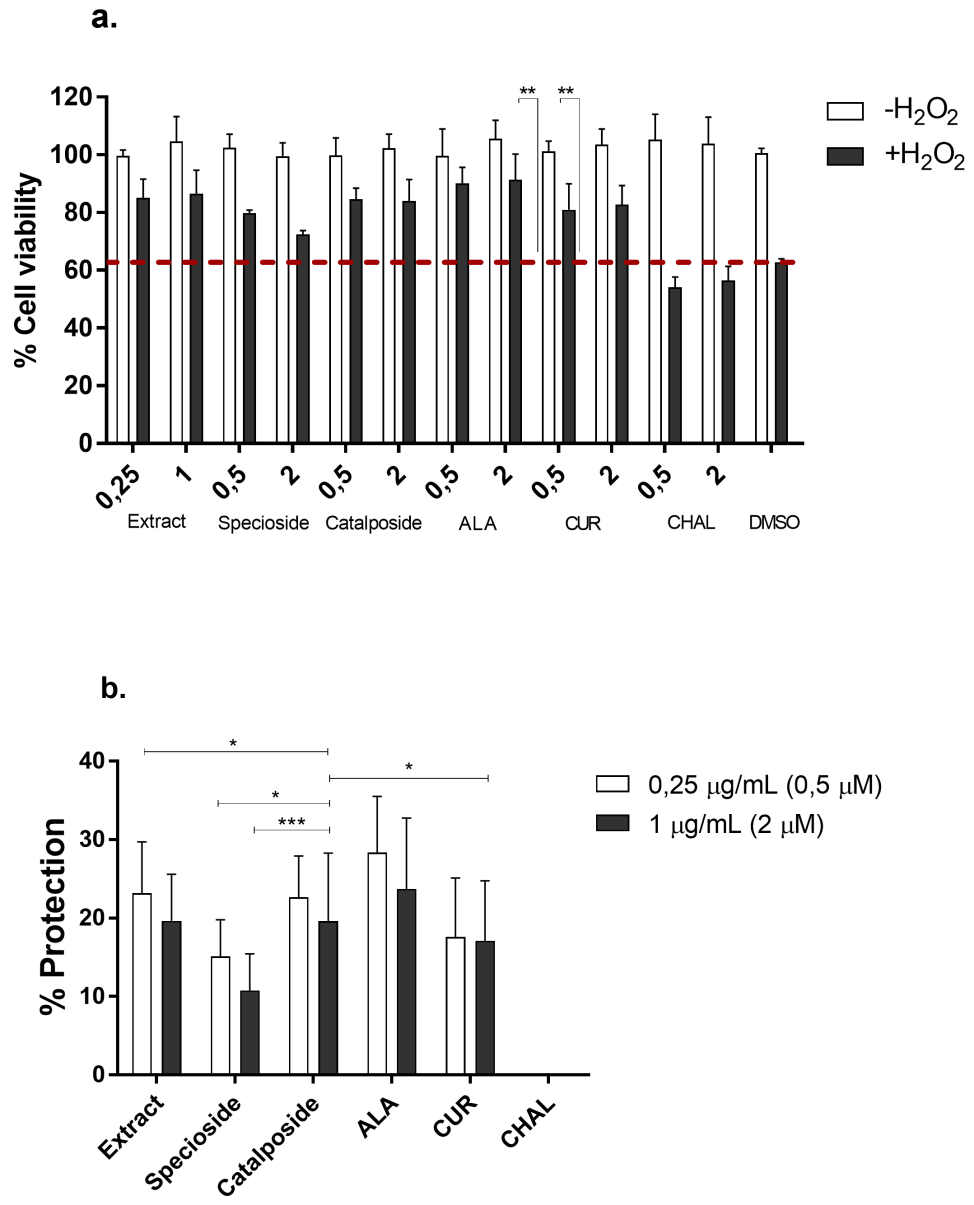
Effect of the *n*-butanol extract, pure compounds and controls on the HepG2 cell line against H _2_O _2_-induced oxidative stress. **a**. Percentage of cell viability. **b**. Protective effect. Kruskal-Wallis, Dunn's post hoc. * p<0.05, ** p<0.01, *** p<0.001. ALA, α-lipoic acid; CUR, curcumin; CHAL, 2-trifluoromethyl-2ʹ-methoxychalcone.

The protective effects of the *n*-butanol extract and the pure compounds against H _2_O _2_-induced oxidative stress was evaluated after 24 hours of exposure to H _2_O _2_. The results showed that exposure to the *n*-butanol extract and pure compounds reduced cell viability to 60 – 70% after H _2_O _2 _exposure ( Figure 6a). A significant protective effect ( *p*<0.05) was observed with the *n*-butanol extract (0.25 µg/mL) and specioside (0.5 and 2 µM), greater than 10%, compared to the control CHAL (2 µM). Additionally, exposure of cells to catalposide at the same concentration (2 µM) displayed a higher protective effect than that of the control CUR and specioside ( Figure 6b). These results indicate that the *n*-butanol extract, specioside, catalposide, ALA and CUR induced protective effects in HepG2 cells against H _2_O _2_-induced oxidative stress at 0.98 mM.

## Discussion

Oxidative stress is the result of an imbalance between the production of reactive oxygen species (ROS) and the cellular antioxidant capacity and plays a critical role in the development of different neurodegenerative diseases and cancer ^12, 44^. Plants are an important source of biologically active natural products, many of which are also models for the synthesis of drugs. Compounds in nature reveal great diversity in terms of chemical structure and biological properties ^45^. Studies carried out by Ghosh *et al*. and Fitmawati *et al*. concluded that medicinal plants are the best sources of phytochemicals and bioactive compounds that are useful for the development of drugs, antioxidants and those showing antidegenerative effects ^46, 47^. Several natural compounds exert their antioxidant effects through the activation of the key transcriptional regulation mechanism of Nrf2, allowing the coordinated expression of antioxidant enzymes such as NQO1 and HMOX-1. The modulation of the Keap1-Nrf2 pathway is important to maintain the homeostatic balance of the cell ^48^.

The antioxidant activity results from the *n-*butanol extract by the ORAC method, which measures the oxygen radical scavenging capacity, showed a concentration-dependent relationship, obtaining the best activity (p <0.05) at 2 µg/mL. There are no reports in the literature regarding the evaluation of the antioxidant capacity of *T. rosea* extracts using the ORAC technique. Studies carried out to determine the hydroxyl scavenging radical capacity from species in the *Tabebuia* genus include those from the crude extracts of the leaves from *Tabebuia chrysantha* G. Nicholson. These studies indicated that the methanolic and aqueous extracts have a significant effect on the uptake of hydroxyl radicals (between 57–86%). Moreover, it was detected that the extracts of *T. chrysantha* can also act to decrease the production of these radicals ^49^. The antioxidant activity reported by Kwak *et al*. for iridoid glycosides showed that specioside and catalposide had potent antioxidant activity by the ORAC method ^50^. When evaluating the antioxidant capacity by the DPPH method, it was observed that at 0.25–2 µg/mL (0.5–4 µM pure compound), an inhibition percentage of 50% was not obtained, and specioside and CUR showed concentration-dependent behavior. The results indicated that the extract of the inner bark of *T. rosea* and the pure compounds show the absence of the DPPH radical scavenging activity. Franco Ospina *et al*. reported the low antioxidant activity of the extracts from the inner bark of *T. rosea* using the DPPH assay. A study of the methanolic extract from the flowers, leaves, roots and inner bark of *T. pallida* showed antioxidant potential using the FRAC and DPPH methods ^51^. Comparative analysis of the ethanolic extracts of *T. rosea* from flowers shows strong antioxidant activity against DPPH and hydroxyl radicals. On the other hand, the report by Joubouhi *et al*. on iridoid compounds found radical scavenging ability against DPPH and ABTSˑ ^+^^52^.

Along with the previous results, the *in vitro* antioxidant activity was carried out with 0.25 and 1 µg/mL extract and 0.5 and 2 µM pure compounds. Activation of the Keap1-Nrf2 pathway revealed the ability of the extract and compounds (specioside and catalposide) to activate this transcription factor, with an increase in the levels of the protein in the nucleus after treatment with the extract and pure compounds. Several natural antioxidant compounds, such as curcumin, sulforaphane and resveratrol, have been reported as electrophilic regulators of the activation of the Keap1-Nrf2 complex. In addition, they are also used for the treatment of different pathologies, such as type 2 diabetes, asthma, and cancer ^53^. The translocation of Nrf2 to the nucleus allows the expression of antioxidant response genes such as *HMOX-1* and *NQO1*. The results show how the compounds and extract increase the expression levels of *HMOX-1* and *NQO1*. A study carried out by our research group evaluated the antioxidant capacity of the ethyl acetate extract from the inner barks of *T. rosea* and *T chrysantha*, in which the capacity of the extracts to activate Nrf2 translocation (after 4 hours of exposure) was reported to induce the expression of *NQO1* ^21^. Glycosylated iridoid compounds such as aucubin ^54^, catalposide and verproside (the main compounds found in the ethyl acetate fraction of *Veronica ciliata*) ^55^ showed a protective effect mediated by Nrf2, increasing the expression levels of the gene and the antioxidant protein NQO1. ** Ma *et al*. reported that aucubin positively regulates Nrf2 translocation and induces the response of phase II antioxidant enzymes such as HMOX-1, NQO1 and SOD, considering aucubin a promising candidate to prevent oxidative stress that induces testicular damage ^56^. Moon *et al*. evaluated the ability of catalposide to induce the expression of *HMOX-1* and its protein in a concentration- and time-dependent manner and found that exposure of neuro-2A cells to catalposide generates a protective effect against H _2_O _2_-induced oxidative stress, increasing the levels of the enzyme HMOX-1 ^25^.

In order to evaluate the protective effects of the extract and compounds, the concentration of H _2_O _2_ (0.98 mM) that induced death by oxidative stress in 50% of the cells was determined. The concentration found in this study for the HepG2 cell line is similar to previous reports ^57^. The results show that the extract and compounds exert a protective effect against oxidative stress induced by H _2_O _2_. The extract, specioside and catalposide had protective effects of more than 10%. The results obtained for catalposide are similar to those reported by Moon *et al*. Catalpol, another glycosylated iridoid, showed a protective effect against oxidative stress induced by H _2_O _2_ in a primary astrocyte culture ^58^. Wang *et al*. evaluated the Nrf2-mediated neuroprotective capacity of swertiamarin, a glycosylated secoiridoid, and reported increases in the levels of the antioxidant proteins NQO1 and HMOX-1 in addition to an increase in the Nrf2 protein at the nuclear level ^59^. The only report of the protective effects of specioside was made by Asthana *et al*., in which the ability of the compound to modulate antioxidant enzymes such as CAT and SOD was evaluated in a *Caenorhabditis elegans* model ^60^.

This study is the first report of the *in vitro* protective effects of the extract of the inner bark of *T. rosea* against oxidative stress induced by H _2_O _2_.

## Conclusion

The present study indicates that the *n*-butanol extract from the inner bark of *T. rosea* and its isolated compound specioside have promising antioxidant activity. Both biocompounds have the ability to activate the Keap1-Nrf2 pathway, inducing the expression of *HMOX-1* and *NQO1*, and generating a protective effect against H _2_O _2_-induced oxidative stress in the HepG2 cell line. These results reinforce the importance of these plants in the search for new antioxidant molecules.

## Data availability

### Underlying data

Open Science Framework: Activation of the Keap1-Nrf2 pathway Tabebuia. https://doi.org/10.17605/OSF.IO/HW6X9 ^43^.

This project contains the following underlying data:

- Dataset 1 Nrf2 Levels Figure 2.csv
- Dataset 2 Nrf2 Levels Figure 3.csv
- Dataset 3 Relative HMOX-1 and NQO1 mRNA levels Figure 4.csv
- Dataset 4 Relative HMOX-1 and NQO1 mRNA levels Figure 5.csv
- Dataset 5 Relative HMOX-1 and NQO1 mRNA levels Figure 6.csv

### Extended data

Open Science Framework: Garzon_et_al_Nrf22020_Supplementary .pdf. https://doi.org/10.17605/OSF.IO/TRVB2 ^23^.

Data are available under the terms of the Creative Commons Zero "No rights reserved" data waiver (CC0 1.0 Public domain dedication).
