# Supplementary material for: Activation of the Keap1-Nrf2 pathway by specioside and the n-butanol extract from the inner bark of Tabebuia rosea (Bertol) DC
Source: F1000Res. 2020 Dec 2;9:1262. Originally published 2020 Oct 21. [Version 3] doi: 10.12688/f1000research.26901.3 (PMC7653643; doi:10.12688/f1000research.26901.3)
Supplement: Peer review history

# Peer review: Reviewer response for version 3

**DOI:** 10.5256/f1000research.31026.r75623

**Published:** 2020-12-03

**Authors:** Luca Rastrelli

**Affiliations:** 1 Department of Pharmacy, University of Salerno, Fisciano, Italy

The authors corrected and clarified the required revisions. They welcomed the criticisms and suggestions. In my opinion this work merits being indexed.

Is the work clearly and accurately presented and does it cite the current literature?

Yes

If applicable, is the statistical analysis and its interpretation appropriate?

Yes

Are all the source data underlying the results available to ensure full reproducibility?

Yes

Is the study design appropriate and is the work technically sound?

Yes

Are the conclusions drawn adequately supported by the results?

Yes

Are sufficient details of methods and analysis provided to allow replication by others?

Yes

Reviewer Expertise:

Phytochemistry, food chemistry

I confirm that I have read this submission and believe that I have an appropriate level of expertise to confirm that it is of an acceptable scientific standard.

# Peer review: Reviewer response for version 1

**DOI:** 10.5256/f1000research.29707.r73587

**Published:** 2020-11-09

**Authors:** Luca Rastrelli

**Affiliations:** 1 Department of Pharmacy, University of Salerno, Fisciano, Italy

The present study indicates that the *n*-butanol extract from the inner bark of *T. rosea *and its isolated compound specioside have promising antioxidant activity.

 This work is well presented and easy to read. The experiments and the structural characterization have been conveniently described and the analyses were performed by appropriate methods. There is sufficient discussion of the results obtained. It merits being indexed after minor revision. Detailed remarks on the text are as follows:

- Extraction and isolation: The authors obtain 12.5 g of BuOH extract, please report the quantities of bark extracted to calculate the yield.
- Figure 1: Report catalposide structure.

Is the work clearly and accurately presented and does it cite the current literature?

Yes

If applicable, is the statistical analysis and its interpretation appropriate?

Yes

Are all the source data underlying the results available to ensure full reproducibility?

Yes

Is the study design appropriate and is the work technically sound?

Yes

Are the conclusions drawn adequately supported by the results?

Yes

Are sufficient details of methods and analysis provided to allow replication by others?

Yes

Reviewer Expertise:

Food chemistry, natural products, analytical chemistry

I confirm that I have read this submission and believe that I have an appropriate level of expertise to confirm that it is of an acceptable scientific standard.

# Response

**Published:** 2020-11-10

**Authors:** Juan Carlos Sepúlveda-Arias

**Affiliations:** Universidad Tecnológica de Pereira, Colombia

Dear Dr. Rastrelli. Thank you very much for your comments. In the "Plant material, extract preparation and specioside isolation" section, we did include the quantity of bark extracted and the yields.

 In Figure 1 we did include the catalposide structure.

# Peer review: Reviewer response for version 1

**DOI:** 10.5256/f1000research.29707.r73506

**Published:** 2020-10-27

**Authors:** Jaime Martin‐Franco

**Affiliations:** 1 Grupo de investigación Química de Productos Naturales QPN, Departamento de Química, Universidad del Cauca, Popayán, Colombia

Plants are an important source of biologically active natural products, many of them also constitute models for the synthesis of numerous drugs. The genus *Tabebuia *includes about 100 species and is the largest genus in the Bignoniaceae family. It is important to indicate that molecules present in the inner bark of some species of this genus (used in traditional medicine) have important pharmacological potential. A large number of chemical compounds exert their antioxidant effects through the activation of key transcriptional regulatory mechanisms, such as the transcription factor Nrf2 (nuclear factor erythroid 2-related factor 2). In cells exposed to oxidative stress. Nrf2 is released and translocated to the nucleus and activates the antioxidant response. The aim of this study was to evaluate the mechanism responsible for the in vitro antioxidant activity of the n-butanol extract obtained from the inner bark of *Tabebuia rosea *(Bertol) DC.

 Specioside was isolated and elucidated previously in the Research Group Polifenoles at Universidad Tecnológica de Pereira from the n-butanol extract obtained from the inner bark of *T. rosea*. The antioxidant activity of specioside was evaluated using the ORAC and DPPH techniques. The effect of specioside and the extract on the viability of HepG2 cells was determined using the MTT method. The effect of the compound and the extract on the translocation of Nrf2 to the nucleus was evaluated using the Nrf2 Transcription Factor Assay Kit (abcam), according to the manufacturer's instructions. Molecular docking was carried out using the AutoDock software.

 The antioxidant activity indicated that both the extract and the isolated compounds have antioxidant activity as measured by the ORAC (oxygen radical absorbance capacity) technique. The cell viability was determined using the MTT assay. The results indicate that specioside exhibits its activity at low concentrations without affecting the viability of the cells since viability was greater than 90% after 24 hours of exposure. They allowed performing *in vitro* tests to evaluate the effect of the isolated compounds on the activation of the Nrf2 signaling pathway in HepG2 cells. Nrf2 is dissociated from Keap1 by the n-butanol extract at a concentration of 0.5 μM after 4 hours of exposure, showing an increase in both cytosolic and nuclear Nrf2 levels with a significant difference (p <0.05) in comparison to the basal levels. Specioside at a concentration of 2 μM did increase the Nrf2 levels in the cytoplasm after 4 hours of exposure and was stabilized in the nucleus at 12 hours after stimulation with specioside and extract (significant difference when compared to the basal levels, p <0.05). The molecular docking analysis showed the interaction of the iridoid group of the ligand with the residue Tyr572 and Ser602 of Keap1 to be important for the interaction with Nrf2.

 In the analysis of the 1H NMR spectrum of the specioside shown in Fig. 1. the authors should write E instead of trans and configuration instead of conformation.

Is the work clearly and accurately presented and does it cite the current literature?

Yes

If applicable, is the statistical analysis and its interpretation appropriate?

Yes

Are all the source data underlying the results available to ensure full reproducibility?

Yes

Is the study design appropriate and is the work technically sound?

Yes

Are the conclusions drawn adequately supported by the results?

Yes

Are sufficient details of methods and analysis provided to allow replication by others?

Yes

Reviewer Expertise:

Organic Synthesis, natural products and spectral analysis by RMN

I confirm that I have read this submission and believe that I have an appropriate level of expertise to confirm that it is of an acceptable scientific standard.

# Response

**Published:** 2020-11-10

**Authors:** Juan Carlos Sepúlveda-Arias

**Affiliations:** Universidad Tecnológica de Pereira, Colombia

Dear Dr. Martin-Franco, Thank you very much for you comments. We did change " *trans* conformation" with " *E* configuration".
